# Comparing patient global impression of severity and patient global impression of change to evaluate test–retest reliability of depression, non-small cell lung cancer, and asthma measures

**DOI:** 10.1007/s11136-022-03180-5

**Published:** 2022-07-19

**Authors:** Sonya Eremenco, Wen-Hung Chen, Steven I. Blum, Elizabeth Nicole Bush, Donald M. Bushnell, Kendra DeBusk, Adam Gater, Linda Nelsen, Stephen Joel Coons, On behalf of the PRO Consortium’s Communication Subcommittee

**Affiliations:** 1 Critical Path Institute, 1730 East River Road, Suite 200, Tucson, AZ USA; 2 GlaxoSmithKline, Collegeville, PA USA; 3 Bristol Myers Squibb, Lawrenceville, NJ USA; 4 Eli Lilly and Company, Indianapolis, IN USA; 5 Evidera | PPD, Phoenix, AZ USA; 6 Seagen Inc, Bothell, WA USA; 7 Adelphi Values, Bollington, Cheshire UK

**Keywords:** Patient-reported outcome measure, Clinical outcome assessment, Test–retest reliability, PGIS, PGIC

## Abstract

**Purpose:**

Score reproducibility is an important measurement property of fit-for-purpose patient-reported outcome (PRO) measures. It is commonly assessed via test–retest reliability, and best evaluated with a stable participant sample, which can be challenging to identify in diseases with highly variable symptoms. To provide empirical evidence comparing the retrospective (patient global impression of change [PGIC]) and current state (patient global impression of severity [PGIS]) approaches to identifying a stable subgroup for test–retest analyses, 3 PRO Consortium working groups collected data using both items as anchor measures.

**Methods:**

The PGIS was completed on Day 1 and Day 8 + 3 for the depression and non-small cell lung cancer (NSCLC) studies, and daily for the asthma study and compared between Day 3 and 10. The PGIC was completed on the final day in each study. Scores were compared using an intraclass correlation coefficient (ICC) for participants who reported “no change” between timepoints for each anchor.

**Results:**

ICCs using the PGIS “no change” group were higher for depression (0.84 vs. 0.74), nighttime asthma (0.95 vs. 0.53) and daytime asthma (0.86 vs. 0.68) compared to the PGIC “no change” group. ICCs were similar for NSCLC (PGIS: 0.87; PGIC: 0.85).

**Conclusion:**

When considering anchor measures to identify a stable subgroup for test–retest reliability analyses, current state anchors perform better than retrospective anchors. Researchers should carefully consider the type of anchor selected, the time period covered, and should ensure anchor content is consistent with the target measure concept, as well as inclusion of both current and retrospective anchor measures.

**Supplementary Information:**

The online version contains supplementary material available at 10.1007/s11136-022-03180-5.

## Plain English Summary

When testing patient-reported outcome (PRO) measures, the ability of the measure to produce the same score over time in respondents with stable health is important. A good PRO measure should give you the same score when someone feels the same and should give you a different score when they feel different. The ability of a measure to produce similar scores is assessed by a statistical test called “test-retest reliability.” This means when you give the same test over again to a group of people who feel the same as they did before, you should get the same score. Sometimes it can be difficult to identify which people feel the same, especially if you have a disease or condition where the symptoms can change a lot. The research team looked at two different ways to identify whether people’s health has changed: a patient global impression of severity (PGIS), where patients answer how they feel at that time, and a patient global impression of change (PGIC), where patients answer if they feel different from another time in the past. In order to see which measure (PGIS or PGIC) works better, the researchers used data that was collected in some other studies. They found that the PGIS does a better job finding patients who feel the same each time and recommend using it when trying to see if a PRO measure can produce consistent results over time. Even though the PGIS worked better, the researchers still suggest also using the PGIC when possible.

## Introduction

Critical Path Institute (C-Path) established the Patient-Reported Outcome (PRO) Consortium in 2008 [1]. As part of supporting the qualification of PRO measures and other clinical outcome assessments (COAs), one of its objectives is to advance the science underpinning the fit-for-purpose assessment of clinical outcomes in trials.

Hence, while the PRO Consortium’s working groups are generating evidence required for the qualification of their respective COAs, additional steps are being taken to gather data that can inform measurement and/or methodological questions. One such question is whether there is an empirical basis for using either static measures of current state (e.g., patient global impression of severity [PGIS]) or retrospective measures of change (e.g., patient global impression of change [PGIC]), to identify stable participants for evaluation of reproducibility of PRO measures.

FDA’s guidance for PRO measures [2] emphasizes the importance of demonstrating reproducibility of scores as one of the measurement properties of a fit-for-purpose PRO measure, commonly assessed by test–retest reliability. In addition, the FDA COA Qualification Program requires documentation of test–retest reliability to support qualification submissions [3]. The purpose of test–retest reliability is to assess the stability of scores over time, i.e., reproducibility. Because scores are expected to change over time due to the participant’s underlying condition changing with an effective intervention, it is necessary to evaluate test–retest reliability with a group of stable participants where no change in condition is observed or reported. One approach is to evaluate test–retest reliability between 2 very close timepoints, and another approach is to use data from the placebo group or from a non-interventional study where change is unlikely to happen. However, each approach has drawbacks. Too short a time interval is subject to “memory effects” where participants remember their responses at timepoint 1 and simply give those same responses at timepoint 2. Whereas in the placebo group or non-intervention approach, participants may still experience change in their condition even without active treatment, especially in conditions where the symptoms are variable. Similar concerns were raised by Reeve and colleagues [4], specifically for multi-item measures. However, in addition to being required for FDA’s COA qualification process, we believe that assessing test–retest reliability provides value when conducted under sufficiently optimal circumstances.

To overcome these drawbacks, selection of the time interval between test and retest should take into consideration symptom variability such that it is long enough to reduce memory effects but short enough that there are a sufficient number of participants whose condition remains stable. Furthermore, to ensure that only stable participants are included in the evaluation of test–retest reliability, anchor measures should be used to identify participants whose condition has not changed during that interval. Anchor measures should assess concepts that are the same or closely related to the concept of interest of the PRO measure in question. Often, patient global assessments are used, especially if there are no other suitable anchor measures.

Two widely used patient global assessments are variations of the PGIC and the PGIS. A PGIC is administered at the end of the test–retest period and asks respondents to rate their symptom severity now/today compared to the beginning of the test–retest period [5]. PGIC response options typically use a bi-directional response scale, e.g., 5 to 7 options ranging from “Much better” to “Much worse” with “No change” at the midpoint. Respondents selecting “No change” form the stable/unchanged subgroup used for test–retest analysis. In contrast, a PGIS asks respondents to rate their symptom or disease severity at a given time, and it is administered at multiple timepoints, including the beginning and the end of the test–retest period. Response options, in the form of a unidirectional verbal rating scale (VRS), often range from “None” to “Very severe.” The stable subgroup used for test–retest analysis is then identified as those respondents providing the same severity rating at those timepoints used in the test–retest analysis. However, there is no consensus or empirical data on which anchor measure performs better in identifying a stable subgroup of participants for the purpose of assessing test–retest reliability. While others have examined the suitability of PGIS and PGIC in other contexts, the authors are not aware of prior studies which compare these anchors specifically within the context of test–retest analysis.

To provide empirical evidence comparing the PGIC and PGIS for identifying a stable subgroup for test–retest analyses, we evaluated data from 3 different working groups within the PRO Consortium, which collected data using both anchors. The Depression Working Group, Non-Small Cell Lung Cancer (NSCLC) Working Group, and Asthma Working Group each conducted independent quantitative pilot studies for developmental versions of their novel PRO measures. The objective of this manuscript is to examine the performance of the PGIS and PGIC in these 3 studies in evaluating test–retest reliability and provide recommendations on their use in examining the stability of a PRO measure’s responses during psychometric evaluation.

## Methods

This section briefly describes methods used to compare the PGIS and PGIC as part of the quantitative pilot studies conducted by each working group during the development of their respective PRO measures.

### Depression study

The *Symptoms of Major Depressive Disorder Scale* (*SMDDS*) is a 16-item measure developed by the Depression Working Group for assessing symptoms of major depressive disorder (MDD) in adults using a 7-day recall period. Each item has a 5-level VRS of either “Not at all/A little bit/Moderately/Quite a bit/Extremely” or “Never/Rarely/Sometimes/Often/Always” depending on whether the item assesses intensity or frequency. Evidence supporting its unidimensionality has been published [6].

The quantitative pilot study included a non-randomized purposive sampling target of 200 participants with clinician-diagnosed MDD recruited from 12 clinical sites within the United States (US) [6]. Participants provided consent via a website and completed a series of web-based measures using a personal computer outside the clinic setting at 2 different timepoints. On Day 1, participants completed the *SMDDS*, a single-item PGIS, and demographic information. Between Days 7 and 10 (hereafter denoted as Day 8), participants completed the same set of measures as Day 1, plus an additional single-item PGIC.

The PGIS asked the following: “How would you rate your depression at this time?” with response options of: “Not depressed,” “Mildly depressed,” “Moderately depressed,” “Very depressed,” and “Extremely depressed.” Positive PGIS change (1, 2, 3) indicates improvement, while negative PGIS change (−1, −2, −3) indicates worsening, in all 3 studies. The PGIC asked participants “Compared to seven days ago, would you describe your depression as…” with the following 7 response options: “Much better,” “Better,” “A little better,” “No change,” “A little worse,” “Worse,” and “Much worse.”

### NSCLC study

The *Non-Small Cell Lung Cancer Symptom Assessment Questionnaire* (*NSCLC-SAQ*) is a 7-item assessment of symptom severity in adults with NSCLC that covers 5 domains (i.e., cough, pain, dyspnea, fatigue, and appetite) and has a 7-day recall period. Each item has a 5-level VRS from either “No < symptom > at All” to “Very severe < symptom > ” or from “Never” to “Always,” depending on whether the item assesses intensity or frequency. Evidence supporting its unidimensionality has been published [7].

The quantitative pilot study included a non-randomized purposive sampling target of 150 participants with clinician-diagnosed NSCLC recruited from 14 US clinical sites [7]. Participants provided consent at the initial clinic visit and completed measures on a touchscreen-enabled tablet computer at their clinic sites at Day 1 and again 7 to 10 days later.

On Day 1, each participant completed the *NSCLC-SAQ*, a single-item PGIS, and demographic information. On Day 8 (accepted within Day 7–10 window), participants returned to the clinic to complete the same measures as Day 1, plus a single-item PGIC.

The PGIS asked the following: “How would you rate your symptoms of your lung cancer at this time?” with response options of: “Not severe,” “Mildly severe,” “Moderately severe,” “Very severe,” and “Extremely severe.” The PGIC asked the following: “Compared to your first study visit, would you describe the symptoms of your lung cancer today as: Much better, Better, A little better, No change, A little worse, Worse, Much worse.”

### Asthma study

The *Asthma Daytime Symptom Diary* (*ADSD*) and the *Asthma Nighttime Symptom Diary* (*ANSD*) are daily assessments of asthma symptom severity appropriate for use in adults and adolescents. The 6-item *ADSD* asks participants to rate each asthma symptom “at its worst since you got up this morning.” The 6-item *ANSD* includes the same symptoms with the timeframe “since you went to bed last night.” Evidence supporting their unidimensionality has been published [8].

The quantitative pilot study was a multi-center, observational study in which PRO data were collected directly from a sampling target of 200 people with asthma recruited from 11 US sites [8]. Participants consented and completed the *ADSD* and *ANSD* daily over 10 days on a provisioned smartphone.

Participants completed the PGIS twice on each study day, once in the morning and once in the evening. The PGIS asked the following: “Overall, please rate your asthma symptoms since you went to bed last night” (*ANSD*) and “Overall please rate your asthma symptoms since you got up this morning” (*ADSD*). The response scale was a 0 to 10 numeric rating scale (NRS) of 0 = “no symptoms” to 10 = “symptoms as bad as you can imagine.”

Participants were asked to complete the PGIC only on Day 10 after they had completed all other study measures. The PGIC asked the following: “Compared to seven days ago, would you describe your asthma symptoms today as: Much better, Better, A little better, No change, A little worse, Worse, Much worse.”

### Test–retest reliability samples

In each case, we provide results for the primary analyses of participants who were designated as stable based on no change in status according to either PGIC or PGIS, as well as exploratory analyses of the full sample of study participants and the subgroup who had no change on both the PGIS and PGIC. These exploratory analyses provide context for comparing the performance of the subgroups identified with the PGIS or PGIC against the unrestricted full sample as well as the most conservative sample. We hypothesized that the PGIS would perform better than the PGIC in these 3 samples because of concerns that recall bias in the case of the retrospective PGIC would result in weaker correlations between change reported on the anchor compared to actual change on the measure itself. Two criteria were used to judge which approach is better: first, the approach needed to yield an ICC of 0.70 or greater, and second, in cases where both approaches exceeded this threshold, the ICCs were compared in terms of which was higher. We also hypothesized that the most conservative sample would yield the highest test–retest reliability as we have more confidence that this subgroup is stable as no change was reported for both anchors. Finally, we hypothesized that the full sample would have the lowest test–retest reliability as it includes participants who have changed between the two timepoints.

For the depression and NSCLC studies, test–retest reliability analyses were conducted using Day 1 and Day 8 data restricted to the subgroup of participants whose depression or NSCLC symptoms remained stable during the study period as defined by the same PGIS responses between Day 1 and Day 8 or “No change” response to the Day 8 PGIC.

For the asthma study, test–retest reliability was evaluated among participants whose experience of asthma symptoms was defined as “stable” between Day 3 and Day 10 defined as:

- Participants completing the *ADSD* and *ANSD* at Day 3 and Day 10 with the same daytime PGIS response and same nighttime PGIS response at both timepoints, or
- Participants completing the *ADSD* and *ANSD* at Day 3 and Day 10 reporting “No change” on the PGIC at Day 10.

### Analyses

To explore within-participant concordance for each study, a cross-tabulation was conducted between responses to the PGIC and level of change on the PGIS.

Test–retest reliability was assessed using a two-way mixed-effect analysis of variance model with interaction for the absolute agreement between single scores, which is the ICC model recommended for test–retest analyses by Qin et al. [9] based on Shrout and Fleiss [10] and McGraw and Wong [11]. ICCs range from 0 to 1, with an ICC ≥ 0.7 indicating good test–retest reliability [12]. ICCs were computed using SPSS [13] in the depression and NSCLC studies and using SAS version 9.4 [14] in the asthma study.

## Results

### Depression study

#### Sample sociodemographic characteristics

A total of 207 participants were enrolled, with 147 participants completing the retest measures. Table 1 shows test and retest participants’ sociodemographic characteristics, with minor differences between these groups. Retest participants were 45 years old on average, 70.7% female, 81% White, and 25% Hispanic/Latino. Almost all (95%) had a minimum high school education, and 59% were employed.

**Table 1:** Participants’ sociodemographic characteristics

Variable	SMDDS		NSCLC-SAQ		ADSD/ ANSD	ADSD	ANSD
	Test *N* = 207	Retest *N* = 147	Test *N* = 152	Retest *N* = 148	Test *N* = 219	Retest *N* = 170	Retest *N* = 180
*Age, years*							
Mean (SD) [Range]	45.3 (14.0) [19–66]	45.4 (14.1) [19–65]	64.3 (9.8) [41–85]	64.5 (9.8) [41–85]	25.8 (17.0) [12–74]	26.0 (17.3) [12–74]	25.7 (16.7) [12–64]
*Sex, n (%)*							
Female	152 (73.4)	104 (70.7)	86 (56.6)	83 (56.1)	120 (54.8)	91 (53.5)	98 (54.4)
Male	55 (26.6)	43 (29.3)	66 (43.4)	65 (43.9)	99 (45.2)	79 (46.5)	82 (45.6)
*Hispanic Origin, n (%)*							
Hispanic or Latino	55 (26.6)	37 (25.2)	8 (5.3)	7 (4.7)	76 (34.7)	59 (34.7)	59 (32.8)
Not Hispanic or Latino	151 (72.9)	109 (74.1)	144 (94.7)	141 (95.3)	143 (65.3)	111 (65.3)	121 (67.2)
Missing	1 (0.5)	1 (0.7)	0 (0.0)	0 (0.0)	0 (0.0)	0 (0.0)	0 (0.0)
*Race,* *n* *(%)*							
White	169 (81.6)	120 (81.6)	132 (86.8)	129 (87.2)	98 (44.7)	73 (42.9)	74 (41.1)
Black or African American	25 (12.1)	17 (11.6)	12 (7.9)	11 (7.4)	65 (29.7)	51 (30.0)	55 (30.6)
American Indian or Alaskan Native	1 (0.5)	0 (0.0)	0 (0.0)	0 (0.0)	1 (.5)	1 (0.6)	1 (0.6)
Asian/Native Hawaiian or Other Pacific Islander	5 (2.4)	4 (2.7)	3 (2.0)	3 (2.0)	11 (5.0)	8 (4.7)	11 (6.1)
Other	6 (2.9)	5 (3.4)	5 (3.3)	5 (3.4)	44 (20.1)	37 (21.8)	39 (21.7)
Missing	1 (0.5)	1 (0.7)	0 (0.0)	0 (0.0)	0 (0.0)	0 (0.0)	0 (0.0)
*Highest level of education completed, n (%)*					Adults (n = 89)	Adults (n = 67)	Adults (n = 75)
Less than high school	11 (5.3)	7 (4.8)	24 (15.8)	24 (16.2)	10 (11.2)	8 (11.9)	8 (10.7)
High school graduate	42 (20.3)	31 (21.1)	55 (36.2)	53 (35.8)	20 (22.5)	16 (23.9)	18 (24.0)
Some college	78 (37.7)	54 (36.7)	39 (25.6)	38 (25.7)	20 (22.5)	14 (20.9)	15 (20.0)
College graduate	40 (19.3)	29 (19.7)	25 (16.4)	24 (16.3)	19 (21.3)	13 (19.4)	17 (22.7)
Graduate or professional school	36 (17.4)	26 (17.7)	9 (5.9)	9 (6.1)	19 (21.3)	16 (23.8)	16 (21.3)
Missing	0 (0.0)	0 (0.0)	0 (0.0)	0 (0.0)	1 (1.1)	0 (0.0)	1 (1.3)
*School grade, n (%)*	N/A*	N/A*	N/A*	N/A*	Adolescents (*n* = 130)	Adolescents (*n* = 103)	Adolescents (*n* = 105)
6th grade					15(11.5)	11(10.7)	10(9.5)
7th grade					26(20.0)	22(21.4)	24(22.9)
8th grade					31(23.8)	27(26.2)	25(23.8)
9th grade					14(10.8)	9 (8.7)	13(12.4)
10th grade					14(10.8)	12(11.7)	10(9.5)
11th grade					15(11.5)	11(10.7)	12(11.4)
12th grade					10 (7.7)	5 (4.9)	6 (5.7)
I have graduated					3 (2.3)	3 (2.9)	2 (1.9)
Missing data					2 (1.5)	3 (2.9)	3 (2.9)
*Employment Status,* *n (%)*					Adults (*n* = 89)	Adults (*n* = 67)	Adults (*n* = 75)
Employed full-time for wages	71 (34.3)	51 (34.7)	20 (13.2)	19 (12.8)	54 (60.7)	40 (59.7)	50 (66.7)
Employed part-time for wages	33 (15.9)	23 (15.6)	6 (3.9)	6 (4.1)	15 (16.9)	13 (19.4)	12 (16.0)
Self-employed	18 (8.7)	13 (8.8)	8 (5.3)	8 (5.4)	N/A*	N/A*	N/A*
Out of work	34 (16.4)	26 (17.7)	10 (6.6)	9 (6.1)	4 (4.5)	5 (7.5)	4 (5.3)
Homemaker	9 (4.3)	5 (3.4)	6 (3.9)	6 (4.1)	4 (4.5)	3 (4.5)	3 (4.0)
Student	12 (5.8)	6 (4.1)	1 (0.7)	1 (0.7)	1 (1.1)	0 (0.0)	0 (0.0)
Retired	5 (2.4)	2 (1.4)	74 (48.7)	74 (50.0)	4 (4.5)	1 (1.5)	2 (2.7)
Unable to work	22 (10.6)	20 (13.6)	27 (17.8)	25 (16.9)	4 (4.5)	4 (6.0)	2 (2.7)
Missing	1 (0.5)	1 (0.7)	0 (0.0)	0 (0.0)	3 (3.4)	1 (1.5)	2 (2.7)
*Self-reported health status, n (%)*							
Excellent	8 (3.9)	6 (4.1)	7 (4.6)	7 (4.7)	32 (14.6)	26 (15.3)	28 (15.6)
Very good	35 (16.9)	23 (15.6)	29 (19.1)	29 (19.6)	99 (45.2)	75 (44.1)	80 (44.4)
Good	76 (36.7)	51 (34.7)	51 (33.6)	49 (33.1)	58 (26.5)	44 (25.9)	50 (27.8)
Fair	66 (31.9)	52 (35.4)	41 (27.0)	40 (27.0)	25 (11.4)	18 (10.6)	17 (9.4)
Poor	22 (10.6)	15 (10.2)	24 (15.8)	23 (15.5)	4 (1.8)	4 (2.4)	3 (1.7)
Missing		0 (0.0)		0 (0.0)	1 (0.5)	3 (1.8)	2 (1.1)

*This variable was not collected

*SMDDS* Symptoms of Major Depressive Disorder Scale, *NSCLC-SAQ* Non-Small Cell Lung Cancer Symptom Assessment Questionnaire, *ADSD* Asthma Daytime Symptom Diary, *ANSD* Asthma Nighttime Symptom Diary

Table 2 shows the cross-tabulation of participant responses to the PGIC (Day 8) and the level of change between PGIS Day 1 to Day 8. As indicated by the shading, 48 participants (33%) had no change on both assessments with another 53 participants (36%) having no change on one measure and a 1 category change on the other. A greater number had no change in PGIS (*n* = 93, 64%) compared to PGIC (*n* = 74, 51%).

**Table 2:**
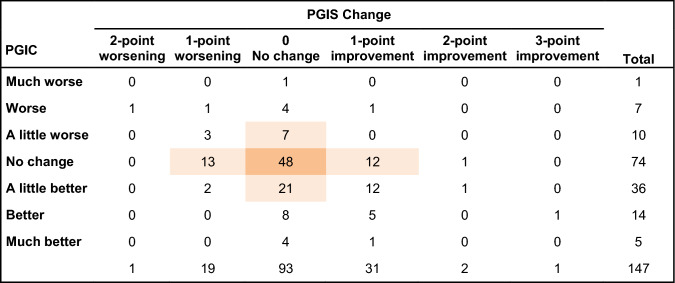
Cross-tabulation of PGIS versus PGIC using SMDDS sample

*PGIC* Patient Global Impression of Change, *PGIS* Patient Global Impression of Severity, *SMDDS* Symptoms of Major Depressive Disorder Scale

No participants indicated having a 3- or 4-point worsening, or a 4-point improvement in PGIS

#### Test–retest reliability

An analysis comparing the PGIS and PGIC was performed by examining the ICC values based on the 3 stable subgroup definitions and the full sample. As shown in Table 3, the ICC of the *SMDDS* score is higher for the PGIS “no change” subgroup (0.84) than it is for the PGIC “no change” subgroup (0.74), although both exceed the recommended threshold of 0.70. Comparisons against the full sample and the most conservative subgroup showed that the PGIS subgroup was almost the same as the most conservative subgroup, while the PGIC subgroup was slightly below the full sample. In this case, the hypothesis that the PGIS subgroup would have a higher ICC than the PGIC subgroup was confirmed, but the full sample and the most conservative subgroup did not perform as hypothesized. The ICCs were similar between the PGIC subgroup and the full sample, and between the PGIS subgroup and the most conservative subgroup.

**Table 3:** Comparison of PGIS vs. PGIC for SMDDS

SMDDS sample or subgroup	ICC	95% Confidence interval
All participants (*N* = 147)	0.755	0.666–0.821
Stable defined by PGIC (N = 74)	0.737	0.607–0.827
Stable defined by PGIS (N = 93)	0.842	0.768–0.893
Stable defined by both PGIS and PGIC (*N* = 48)	0.841	0.709–0.912

*SMDDS* Symptoms of Major Depressive Disorder Scale, *ICC* intraclass correlation coefficient, *PGIC* Patient Global Impression of Change, *PGIS* Patient Global Impression of Severity

Two-Way Mixed with Absolute Agreement (using SPSS)

### NSCLC study

#### Sample sociodemographic characteristics

A total of 152 participants from 14 US sites were enrolled, with 148 participants completing the retest measures. Retest participants were 64 years old on average, 56% female, 87% White, and 5% Hispanic/Latino (Table 1). The majority (84%) had a minimum high school education, 50% were retired, and 17% were unable to work. No differences were noted between test and retest participants.

Table 4 shows the cross-tabulation of NSCLC participants and their responses to the PGIC (Day 8) and the level of change between PGIS Day 1 to Day 8. As indicated by the shading, 56 participants (38%) had no change on both assessments with another 42 participants (28%) deviating by 1 point on either assessment.

**Table 4:**
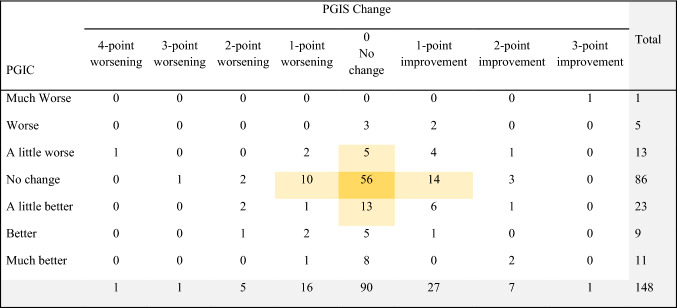
Cross-tabulation of PGIS versus PGIC using NSCLC-SAQ sample

*NSCLC-SAQ* Non-Small Cell Lung Cancer Symptom Assessment Questionnaire, *PGIC* Patient Global Impression of Change, *PGIS* Patient Global Impression of Severity

No participants indicated having a 4-point improvement in PGIS

#### Test–retest reliability

An analysis comparing the PGIS and PGIC using the *NSCLC-SAQ* sample was performed by examining the ICC values based on the 3 stable subgroup definitions and the full sample. As shown in Table 5, the ICC of the *NSCLC-SAQ* score is slightly higher for the PGIS “no change” subgroup (0.87) than the PGIC “no change” subgroup (0.85), although both exceed the recommended threshold of 0.70. Comparisons against the full sample and the most conservative subgroup showed that the ICC values were as hypothesized. The most conservative sample had the highest ICC, followed by PGIS and PGIC subgroups; the full sample had the lowest ICC.

**Table 5:** Comparison of PGIS vs. PGIC for NSCLC-SAQ

NSCLC-SAQ sample or subgroup	ICC	95% Confidence Interval
All participants (*N* = 148)	0.809	0.745–0.858
Stable defined by PGIC (*N* = 86)	0.851	0.780–0.900
Stable defined by PGIS (*N* = 90)	0.866	0.804–0.910
Stable defined by both PGIS and PGIC (*N* = 56)	0.892	0.822–0.935

*NSCLC-SAQ* Non-Small Cell Lung Cancer Symptom Assessment Questionnaire, *ICC* intraclass correlation coefficient, *PGIC* Patient Global Impression of Change, *PGIS* Patient Global Impression of Severity

Two-Way Mixed with Absolute Agreement (using SPSS)

### Asthma study

#### Sample sociodemographic characteristics

A total of 219 participants were recruited from 13 US sites, with 170 completing the *ADSD* on both assessment days, 180 completing the *ANSD* on both assessment days, 168 completing the PGIS nighttime on both assessment days, 166 completing the PGIS daytime on both assessment days, and 180 completing the PGIC on Day 10. Test and retest participants’ sociodemographic characteristics are shown in Table 1, with only minor differences found. Participants were 25 years old on average, 54% female, 41% White, and 35% Hispanic/Latino. The majority of adolescent participants were in 7th (22.9%) or 8th grade (23.8%). The majority of adults (89%) had a minimum high school education, and 67% were working full-time (Table 1).

Table 6 shows the cross-tabulation of participants and their responses to the PGIC (Day 10) and the level of change between PGIS between Day 3 and Day 10 for the *ANSD*. As indicated by the shading, only 14 participants (9.5%) had no change on both assessments, with another 38 participants (25.7%) deviating by 1 point on either assessment. Similar analysis for the PGIS and PGIC on the *ADSD* indicated 25 participants (15.1%) had no change on both assessments, with another 28 participants (17.0%) deviating by 1 point on either assessment (Table 7).

**Table 6:**
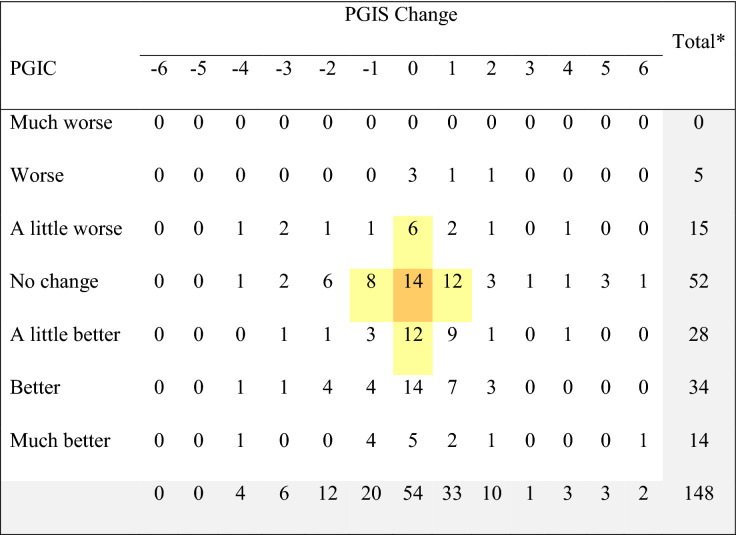
Cross-tabulation of PGIS change between Day 3 and Day 10 versus PGIC ratings for the ANSD

*Total sample for the cross-tabulation includes participants who had both PGIC at Day 10 and PGIS at Days 3 and 10 for the *ANSD*

*PGIS* Patient Global Impression of Severity, *PGIC* Patient Global Impression of Change, *ADSD* Asthma Nighttime Symptom Diary

Positive PGIS change indicates improvement, while negative PGIS change indicates worsening. PGIS used a 0 to 10 numeric rating scale, which means that change could range from − 10 to + 10. No participants indicated having a 7 − , 8 − , 9 −, or 10-point worsening, or a 7 − , 8 − , 9 −, or 10-point improvement in PGIS

**Table 7:**
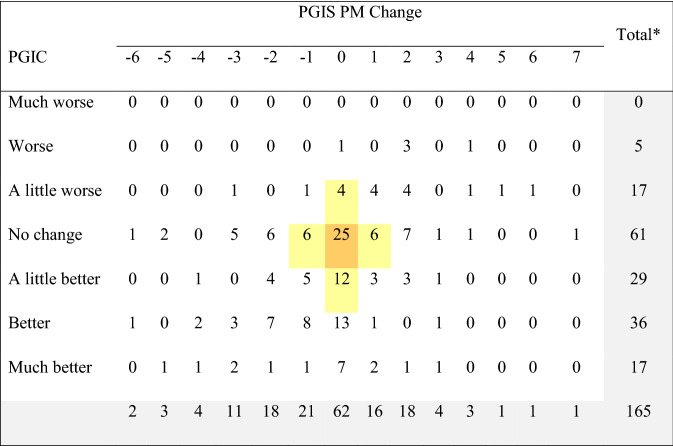
Cross-tabulation of PGIS change between Day 3 and Day 10 versus PGIC ratings for the ADSD

*Total sample for the cross-tabulation includes participants who had both PGIC at Say 10 and PGIS at Days 3 and 10 for the *ADSD*

*PGIS* Patient Global Impression of Severity, *PGIC* Patient Global Impression of Change, *ADSD* Asthma Daytime Symptom Diary

Positive PGIS change indicates improvement, while negative PGIS change indicates worsening. PGIS used a 0 to 10 numeric rating scale, which means that change could range from − 10 to + 10. No participants indicated having a 7 − , 8 − , 9 −, or 10-point worsening, or an 8 − , 9 −, or 10-point improvement in PGIS

#### Test–retest reliability

An analysis comparing the PGIS and PGIC using the *ANSD* and *ADSD* samples was performed by examining the ICC values based on the 3 stable group definitions and the full sample. Scores for both the *ANSD* (Table 8) and *ADSD* (Table 9) demonstrated “good” test–retest reliability (ICC = 0.95 and 0.86, respectively) when participants were defined as “stable” between Day 3 and 10 according to PGIS ratings. When participants were defined as “stable” according to PGIC ratings, lower ICCs indicative of “moderate” test–retest reliability (ICC = 0.53 and 0.68, respectively) were observed. For the *ANSD*, the full sample was higher than the PGIC subgroup, which was not as hypothesized, while for the *ADSD*, the PGIC subgroup had higher ICCs than the full sample (all under 0.70). The PGIS subgroup ICC was almost as high as the most conservative subgroup for the *ANDS* and slightly higher than the full sample for the *ADSD* (all greater than 0.8).

**Table 8:** Comparison of PGIS vs. PGIC for ANSD

ANSD sample or subgroup	ICC	95% confidence interval
All participants (*N* = 180)	0.644	0.549–0.722
Stable defined by PGIC (*N* = 54)	0.533	0.312–0.699
Stable defined by PGIS (*N* = 61)	0.954	0.924–0.972
Stable defined by both PGIS and PGIC (*N* = 14)	0.973	0.915–0.991

*ANSD* Asthma Nighttime Symptom Diary, *ICC* intraclass correlation coefficient, *PGIC* Patient Global Impression of Change, *PGIS* Patient Global Impression of Severity

Two-Way Mixed Effects Model with Absolute Agreement (using SAS)

**Table 9:** Comparison of PGIS vs. PGIC for ADSD

ADSD sample or subgroup	ICC	95% confidence interval
All participants (*N* = 170)	0.595	0.489–0.684
Stable defined by PGIC (*N* = 62)	0.675	0.514–0.790
Stable defined by PGIS (*N* = 63)	0.858	0.776–0.912
Stable defined by both PGIS and PGIC (*N* = 25)	0.843	0.675–0.928

*ADSD* Asthma Daytime Symptom Diary, *ICC* intraclass correlation coefficient, *PGIC* Patient Global Impression of Change, *PGIS* Patient Global Impression of Severity

Two-Way Mixed Effects Model with Absolute Agreement (using SAS)

## Discussion

Results from all 3 case examples indicate that acceptable ICCs were obtained using the PGIS to identify a stable subgroup for evaluating the test–retest reliability of a PRO measure. The PGIS performed better in the depression and asthma studies, while both PGIS and PGIC performed similarly in the NSCLC study. Only the PGIC for the asthma study resulted in an ICC below 0.70. These results suggest that when the symptoms are generally stable across time, both PGIS- and PGIC-based subgroups may yield similar ICC values, whereas when symptoms fluctuate from day-to-day, PGIS is more likely to identify stable participants and yield higher ICCs. This observation is also supported by the ICC results using the full sample and the most conservative subgroup. While the ICCs of the conservative subgroups were always higher than those for the full samples as hypothesized, the difference was greatest for the asthma study where symptom severity was more likely to vary from day-to-day. While the estimated test–retest reliability for the full sample appeared to be numerically higher than the PGIC subgroups which was not as hypothesized, the important message is that they were not within the commonly acceptable range as was the test–retest reliability based on PGIS and the most conservative group.

Overall, these comparisons show that both the PGIS and PGIC were able to identify a stable subgroup for test–retest analyses in 2 of the 3 cases, and researchers may want to include both anchors to generate sufficient evidence to support stability of the subgroup for analysis. Researchers may even consider using the most conservative subgroup to calculate test–retest reliability (i.e., participants identified as stable based on both PGIS and PGIC). However, such a stringent definition may result in a small sample size insufficient for a robust test–retest analysis as was the case for *ADSD*/*ANSD* (*n* = 14 and 25, respectively). In the 3 studies examined in this paper, it appears that using PGIS alone is sufficient to yield results similar to the most conservative sample.

The results observed in the 3 studies may also be affected by the type of global rating scales used. Each study used a similar bi-directional response scale for the PGIC, but the wording of the PGIS rating scale varied. Both the depression and NSCLC studies employed a 5-level VRS, whereas the asthma study employed a 0 to 10 NRS. It is possible that the differences in ICCs among the 3 studies are due to the interaction of the symptom characteristics and the types of rating scales used for the global anchors. The additional response options for the PGIS scale in the asthma study may have created more variability in responses and subsequently noise in the results than the 5-level VRS. We recommend that researchers, when cognitively evaluating a newly developed or existing measure, also consider including proposed anchor measures to ensure that the anchor measures are optimally worded and meaningful to participants, if feasible. At a minimum, we recommend that the anchor measures assess the same or sufficiently similar concepts and have the same recall period as the PRO measure in question.

In the studies reported above, an observational design was used. However, even in an observational design it may be challenging to identify stable participants, because medication use is not controlled. While more than half the participants in the depression and NSCLC studies were considered stable, only a third of the participants in the asthma study were considered stable. This may also depend on the variability of the symptoms included in the measure. Researchers should take this into account when considering the sample size for their studies.

There are a number of limitations associated with our study. This article presents results of secondary analyses of data initially collected for another purpose, and therefore the studies may be underpowered for the comparisons made here. Daily mean scores on the *ADSD*/*ANSD* were compared at the test and retest timepoints, rather than comparing weekly mean scores for each measure. With high symptom variability in asthma where symptoms may fluctuate from day-to-day, even those with stable asthma overall may have different symptom scores on days a week apart. The asthma study used a PGIC with a 7-day recall period, while the scores being compared were the Day 3 and Day 10 scores, which deviates from the recommendation to align the recall period of the anchor measure with the measure in question. The asthma study included adolescents and adults, who may approach global ratings differently resulting in more noise in the data. In addition, because participants were recruited from clinical sites, participants could not be readily replaced if they failed to complete their retest assessment, and we lack information on why they dropped out and whether it was random or not. Therefore, we have no way to evaluate whether dropouts impacted the ICC results. PGIS and PGIC wording and scale formats were inconsistent across the 3 studies reported here, which limits our ability to compare their performance. Finally, this study did not assess test–retest reliability using timepoints more than 7 days apart. There are situations where test–retest analysis timepoints may be less or more than 7 days. How these anchors perform in different time intervals needs to be studied further.

For researchers planning to assess test–retest reliability in observational studies, we offer the following recommendations:

1. Include both the PGIS and PGIC as anchors. While the PGIS performed better in our studies overall, the PGIC can be used to identify a complementary stable subgroup for confirming test–retest reliability. In addition, researchers should also consider the use of other measures, including clinician assessments, as anchors. When multiple anchors are being used, researchers should pre-specify which measure will be used for the primary analysis of test–retest reliability, or how they plan to triangulate results from multiple measures.
2. Researchers need to consider the variability of the individual symptoms that are being assessed with their PRO measure. Some symptoms may be relatively stable from day-to-day, while others may vary a great deal. This may be an important consideration when thinking about assessing health status on a single day or over several days.

While not derived directly from the results of the study, we provide the following recommendations regarding the design and evaluation of anchor measures for use in future research.

1. Researchers should be thoughtful in the design of their anchor measure to ensure that it assesses the same or sufficiently similar concept as the PRO measure in question. Researchers should also consider the recall period being used (e.g., current state as compared to status on a specific day or over a period of time).
2. Researchers should consider including the evaluation of the anchor measures as part of the cognitive interview phase for new PRO measure development or when conducting qualitative research with existing measures. If feasible, this will help to ensure the relevance and comprehension of the anchor measures.

Considerations regarding anchor measure selection and implementing test–retest analyses in interventional studies are provided in the online Supplementary Information.

## Conclusions

Three PRO Consortium working groups employed both retrospective assessment of change using PGIC as well as “current state” assessment of disease or symptom severity using the PGIS to identify a stable subgroup in which to assess the test–retest reliability of their PRO measures in development. PGIS performed better that the PGIC for the depression and asthma studies, and both anchors performed similarly for the NSCLC study. These results provide empirical evidence about the use of current state and retrospective anchor measures within the context of assessing test–retest reliability. In addition, we have provided recommendations for consideration when including these and other anchor measures in the evaluation of test–retest reliability.

## Supplementary Information

Below is the link to the electronic supplementary material.

Supplementary file1 (DOCX 17 kb)

## Data Availability

The data used for the secondary analyses described in this paper were collected in studies funded by the following PRO Consortium member firms: AbbVie, Allergan, AstraZeneca AB, Boehringer Ingelheim, Bristol Meyers Squibb, Eli Lilly and Company, EMD Serono, Genentech, Inc., GlaxoSmithKline, LLC, Janssen Global Services, LLC, Merck Sharp & Dohme Corp., Novartis Pharma AG, Pfizer, Inc., Sanofi, Sunovian Pharmaceuticals, Inc., and Takeda Pharmaceuticals International.

## References

[1] Coons SJ, Kothari S, Monz BU, Burke LB (2011). The Patient-Reported Outcome (PRO) Consortium: filling measurement gaps for PRO endpoints to support labeling claims. Clinical Pharmacology Therapeutics.

[2] Food and Drug Administration Center for Drug Evaluation and Research. Guidance for Industry: Patient-Reported Outcome Measures: Use in Medical Product Development to Support Labeling Claims. Federal Register: December 9, 2009. http://www.fda.gov/downloads/Drugs/GuidanceComplianceRegulatoryInformation/Guidances/UCM193282.pdf. [Accessed January 15, 2021]

[3] Food and Drug Administration Center for Drug Evaluation and Research. FDA COA Full Qualification Package Outline. https://www.fda.gov/media/147025/download [Accessed December 17, 2021]

[4] Reeve BB, Wyrwich KW, Wu AW, Velikova G, Terwee CB, Snyder CF, Schwartz C, Revicki DA, Moinpour CM, McLeod LD, Lyons JC, Lenderking WR, Hinds PS, Hays RD, Greenhalgh J, Gershon R, Feeny D, Fayers PM, Cella D, Brundage M, Ahmed S, Aaronson NK, Butt Z (2013). ISOQOL recommends minimum standards for patient-reported outcome measures used in patient-centered outcomes and comparative effectiveness research. Quality of Life Research.

[5] Jaeschke R, Singer J, Guyatt GH (1989). Measurement of health status. Ascertaining the minimal clinically important difference. Controlled Clinical Trials.

[6] Bushnell DM, McCarrier KP, Bush EN, Abraham L, Jamieson C, McDougall F, Trivedi M, Thase M, Carpenter L, Coons SJ, on behalf of the Patient-Reported Outcome Consortium’s Depression Working Group. (2019). Symptoms of Major Depressive Disorder Scale (SMDDS): performance of a novel patient-reported symptom measure. Value in Health.

[7] Bushnell DM, Atkinson T, McCarrier K, Liepa A, DeBusk K, Coons SJ (2021). Non-Small Cell Lung Cancer Symptom Assessment Questionnaire (NSCLC-SAQ): psychometric performance and regulatory qualification of a novel patient-reported symptom measure. Current Therapeutic Research.

[8] Gater A, Nelsen L, Coon CD, Eremenco S, O’Quinn S, Khan AH, Eckert L, Staunton H, Bonner N, Hall R, Krishnan JA, Stoloff S, Schatz M, Haughney J, Coons SJ, on behalf of the Patient-Reported Outcome Consortium’s Asthma Working Group (2022). Asthma Daytime Symptom Diary (ADSD) and Asthma Nighttime Symptom Diary (ANSD): measurement properties of novel patient-reported symptom measures. The Journal of Allergy and Clinical Immunology: In Practice..

[9] Qin S, Nelson L, McLeod L, Eremenco S, Coons SJ (2019). Assessing test-retest reliability of patient-reported outcome measures using intraclass correlation coefficients: recommendations for selecting and documenting the analytical formula. Quality of Life Research.

[10] Shrout PE, Fleiss JL (1979). Intraclass correlations: Uses in assessing rater reliability. Psychological Bulletin.

[11] McGraw KO, Wong SP (1996). Forming inferences about some intraclass correlation coefficients. Psychological Methods.

[12] Nunnally J, Bernstein IH (1994). Psychometric theory.

[13] SPSS Inc. (2002). SPSS (for Windows). Rel. 11.5.0. SPSS Inc, Chicago, IL.

[14] SAS Institute Inc (2013). SAS Version 9.

