# Supplementary material for: Comparing patient global impression of severity and patient global impression of change to evaluate test–retest reliability of depression, non-small cell lung cancer, and asthma measures
Source: Qual Life Res. 2022 Jul 19;31(12):3501–12. doi: 10.1007/s11136-022-03180-5 (PMC9587936; doi:10.1007/s11136-022-03180-5)
Supplement: Supplementary file 1 — Supplementary file1 (DOCX 17 kb) [file 11136_2022_3180_MOESM1_ESM.docx]

**Supplementary Information**

This supplementary information section contains additional information that may be of interest to readers of the main article and is intended for publication.

**Considerations for Anchor Measures**

It is important to note that although the anchor measures are referred to as PGIC and PGIS, they are not “global” in the sense of assessing the overall experience of the participant related to the condition including symptoms, functional limitations, and impacts on health-related quality of life/quality of life (HRQOL/QOL). Rather, global refers to assessment of the overall status of the concept of interest. In 2 of the examples in this manuscript, it is the overall assessment of disease symptoms, while in the third the overall condition status was assessed. When selecting an anchor, it is important to consider the implications of using an overall HRQOL/QOL anchor, overall disease/condition status anchor, overall disease symptoms anchor, or a specific symptom anchor, as responses may vary depending on the disease or condition and the manifestation of symptoms.

**Considerations for evaluating test-retest reliability within an interventional study**

Additional considerations are needed if evaluating test-retest reliability within an interventional study, such as a randomized clinical trial. Unlike these quantitative pilot studies, where test-retest evaluation was incorporated as part of the study design, in clinical trials, assessments for test-retest reliability will have to fit within the existing visit schedule. As a result, there may be less flexibility in selecting such time points. Given the interventional nature of the study, researchers need to consider the use of medication and the onset of effect. In clinical trials, test-retest reliability may be evaluated between the screening period and randomization, however, a participant may still be washing out of prior treatments during the screening run-in period. Another option is to look at a stable population toward the end of a study (if efficacy stabilizes). The use of electronic data collection, such as via web-based assessments or use of a tablet or smartphone, may allow for additional flexibility of remote assessments as compared to assessments which are collected in the clinic during study visits. Researchers also need to consider the baseline or referent period for the PGIC. In the present studies, we used a comparison to the severity of their symptoms or condition 7 days earlier. It may be preferable to refer to a specific event, such as the start of the study rather than a specific day, as this may be cognitively challenging for respondents. In clinical trials, this can be further complicated by studies or design features such as blinded-run in periods or cross-over periods. However, the complexity and challenge of assessing test-retest reliability in an interventional study further emphasizes the importance of using adequate anchor(s) to identify the subgroup of stable participants.

When used in interventional studies, such as randomized clinical trials, care must be taken when selecting the study visits/timepoints for evaluation of test-retest reliability. Researchers need to consider onset of efficacy with interventional treatment, but also need to consider wash-out periods and the use of concomitant and rescue medications. The use of electronic data collection methods may allow for increased flexibility regarding when such assessments are completed as compared to in-clinic data collection.
